# Charge transport through extended molecular wires with strongly correlated electrons†

**DOI:** 10.1039/d1sc03050g

**Published:** 2021-07-26

**Authors:** James O. Thomas, Jakub K. Sowa, Bart Limburg, Xinya Bian, Charalambos Evangeli, Jacob L. Swett, Sumit Tewari, Jonathan Baugh, George C. Schatz, G. Andrew D. Briggs, Harry L. Anderson, Jan A. Mol

**Affiliations:** Department of Materials, University of Oxford Parks Road Oxford OX1 3PH UK james.thomas@materials.ox.ac.uk; Department of Chemistry, University of Oxford, Chemistry Research Laboratory Oxford OX1 3TA UK; Department of Chemistry, Northwestern University Evanston Illinois 60208 USA; Department of Chemistry, Rice University Houston TX USA; Institute for Quantum Computing, University of Waterloo Waterloo ON N2L 3G1 Canada; School of Physics and Astronomy, Queen Mary University of London London E1 4NS UK

## Abstract

Electron–electron interactions are at the heart of chemistry and understanding how to control them is crucial for the development of molecular-scale electronic devices. Here, we investigate single-electron tunneling through a redox-active edge-fused porphyrin trimer and demonstrate that its transport behavior is well described by the Hubbard dimer model, providing insights into the role of electron–electron interactions in charge transport. In particular, we empirically determine the molecule's on-site and inter-site electron–electron repulsion energies, which are in good agreement with density functional calculations, and establish the molecular electronic structure within various oxidation states. The gate-dependent rectification behavior confirms the selection rules and state degeneracies deduced from the Hubbard model. We demonstrate that current flow through the molecule is governed by a non-trivial set of vibrationally coupled electronic transitions between various many-body ground and excited states, and experimentally confirm the importance of electron–electron interactions in single-molecule devices.

## Abstract 2

Experimental studies of electron transport through an edge-fused porphyrin oligomer in a graphene junction are interpreted within a Hubbard dimer framework.

## Graphical abstract

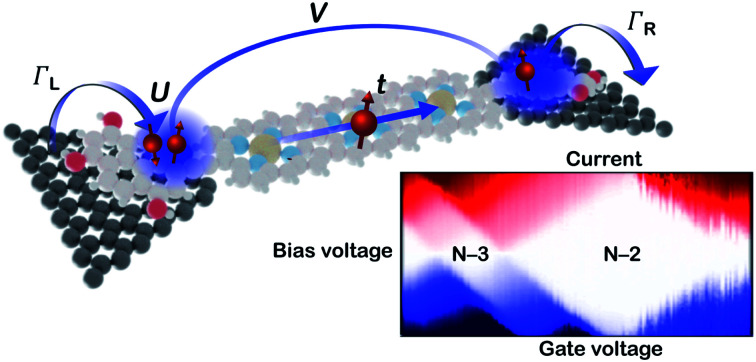

## Introduction

Charge transport is one of the key observables in quantum systems, yet its interpretation is often complicated by strong many-body correlations. In molecular systems, these electron–electron and electron–vibration interactions are especially important in the resonant transport regime, and a rich tapestry of transport and out-of-equilibrium phenomena has been observed in single-molecule junctions.^1–7^ For most single-molecule junctions these phenomena are limited to local interactions, including the observation of Coulomb blockade (and related Pauli blockade) and Franck–Condon blockade. In extended molecular systems, more intricate interacting approaches such as the fermionic Hubbard model that account for electron–electron interactions beyond the observation of Coulomb blockade^8–15^ are important in describing experimental results.^16–18^

The Hubbard model is a ubiquitous description of strongly correlated condensed matter systems, including high-temperature superconductors and topological insulators. From a molecular perspective, the fermionic Hubbard model is an extension to the non-interacting Hückel model, which has been used very successfully in combination with Landauer theory to describe off-resonance quantum transport through extended molecules, but fails in the resonant transport regime where electron–electron interactions become dominant.^19^ By contrast, the Hubbard model not only considers the kinetic ‘hopping’ terms but also accounts for the Coulomb potentials. Under the assumption that the electronic structure can be derived from these interactions between localized sites within a molecular structure, it is an extremely useful tool to empirically parameterize the many-body interactions that make up molecular structure–property relations.

**Fig. 1:**
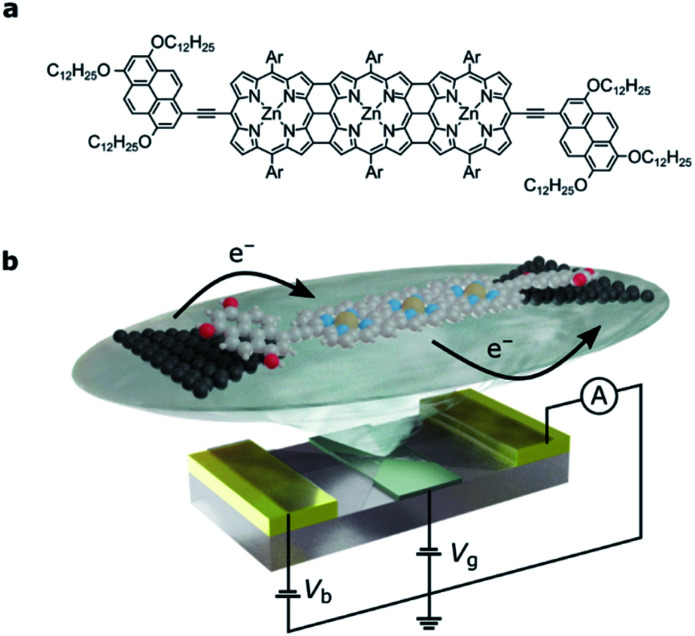
(a) The molecular structure of **FP3**: the edge-fused porphyrin trimer core is functionalized in the terminal *meso* positions by tridodecyloxypyrene groups for anchoring to graphene source and drain electrodes. Ar groups are solubilizing aryl groups, 3,5-bis(trihexylsilyl)phenyl. (b) Device architecture: nanometer-separated graphene source and drain electrodes are electroburnt from a graphene ribbon between two gold electrodes. The graphene is patterned into a bowtie shape, and a local gate electrode separated from the molecule by a thin layer of HfO_2_ (grey) is used to shift the molecular energy levels. For clarity, the bulky side-groups are omitted.

Here, we investigate charge transport through an edge-fused zinc porphyrin trimer, **FP3** (Fig. 1a), that is weakly coupled to graphene source and drain electrodes through two electron-rich pyrene anchor groups. Unlike in most single-molecule devices where only one or two charge-states are accessible,^20,21^ the high redox-activity of this fully conjugated oligomer enables us to study up to four charge-states. This in turn lets us measure the addition energies and out-of-equilibrium current rectification that are a result of electron transfers between the many-body Fock states which arise from partial filling of the two highest occupied molecular orbitals. Due to their close energy spacing in the extended molecular structure, and their symmetry properties, they can be transformed into localized orbitals that have high electron density on either end group of the molecule. The weak molecule–electrode coupling means that electron transport through the whole molecular junction depends on the occupancy of these spatially localized orbitals, so that electronic correlations must be taken into account. We interpret the results in the framework of a Hubbard dimer, the simplest non-trivial Hubbard Hamiltonian, to confirm that the transport properties of **FP3** are dominated by the HOMO and HOMO−1. Consequently, we can quantify the strength of the electron–electron and electron–vibration interactions and determine the Dyson coefficients that correspond to the wavefunction overlap between the Fock states that arise from filling these orbitals.

## Results and discussion

### Molecular devices

We designed the molecule **FP3** such that it contains two electron-rich anchor groups separated by a conjugated edge-fused porphyrin trimer. The three bonds between each porphyrin result in a planar structure and thus enhance electron delocalization across **FP3**.^22–26^ The electrochemical gap of **FP3** from square-wave voltammetry is 0.8 eV (as compared to 1.7 eV for a zinc porphyrin monomer with the same anchor groups^20^). The longest wavelength absorbance maximum in the optical absorbance spectrum of **FP3** is at 1500 nm (0.83 eV), compared to 700 nm (1.77 eV) for the monomer (**FP3** spectra are in the ESI†).

The single-molecule device architecture is shown in Fig. 1b and is described in more detail in the ESI.† Briefly, graphene source and drain electrodes, separated by approximately 1–2 nm, are fabricated by electron-beam lithography and feedback-controlled electroburning.^27,28^ A solution (2 μM in toluene) of **FP3** is drop-cast on the electrodes. The tridodecyloxypyrene (TDP) anchor groups on the periphery of the fused porphyrin unit interact with the graphene electrodes through a π-stacking interaction. They have a calculated binding energy of −3.2 eV to the graphene surface, and as we have shown before, are necessary to achieve a functional molecular device yield. The π-stacking leads to weak molecule–electrode coupling, while the aryl groups (Ar, Fig. 1a) prevent molecular aggregation (see ESI†) and are essential for solubility. The gate electrode is either the doped silicon substrate with a thermally grown 300 nm SiO_2_ dielectric (device **A**, device **B**) or gold with a 10 nm layer of HfO_2_ dielectric grown by atomic-layer deposition (device **C**, and shown in Fig. 1b). Stability diagrams prior to molecular deposition are included in the ESI† and confirm that the signals observed are due to the deposition of **FP3**, and not residual carbon quantum dots from the electroburning process.^20^

### Extended Hubbard model

**Fig. 2:**
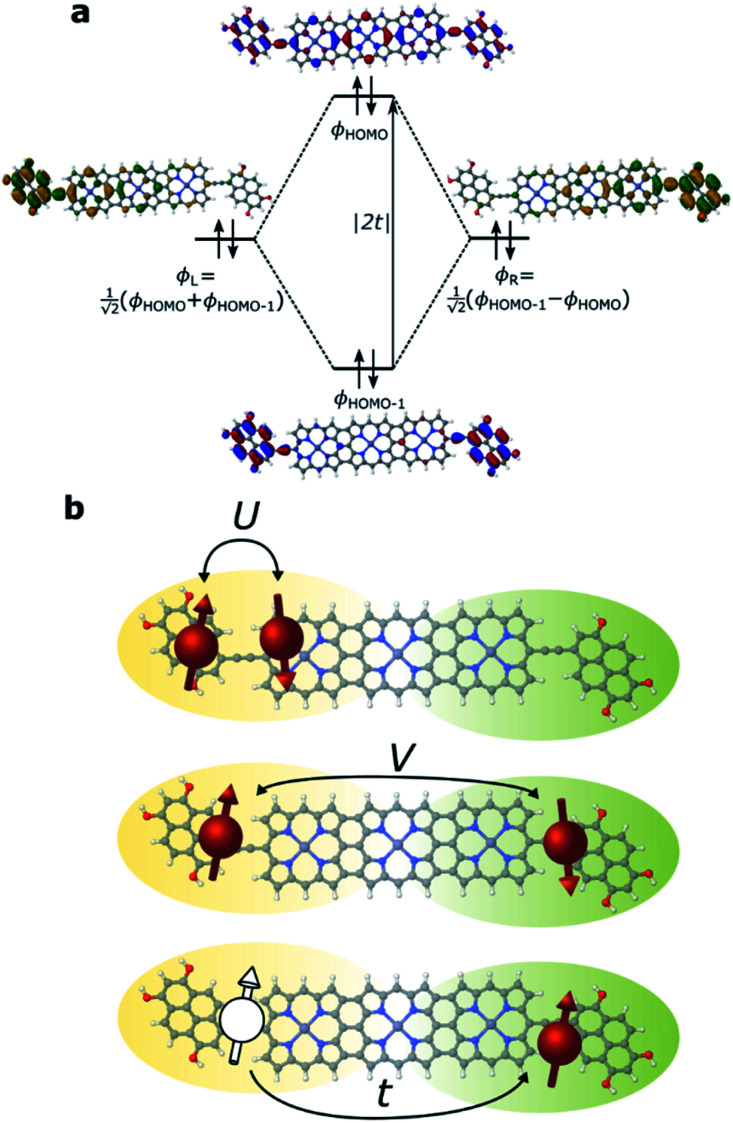
(a) MO diagram displaying the eigenbasis and site basis of the frontier orbitals of **FP3** in the *N* state. Since the sum of the site orbitals yields the MO that is lower in energy the tunnel coupling, *t*, is negative. (b) Visualization of the Hubbard terms for **FP3**, *U* and *V* are potential energy terms due to on-site and inter-site repulsion, and *t* is the kinetic energy term accounting for hopping between localized sites.

We have previously shown that the porphyrin monomer with the same electron-rich TDP anchor groups is commonly found in the oxidized *N* − 1 state (where *N* is the number of electrons on the molecule in the neutral state) upon adsorption onto p*-*doped graphene electrodes at zero gate voltage, *V*_g_ = 0.^29^**FP3** is more readily oxidized when compared to the monomer (first oxidation potentials are −0.07 V and 0.04 V for **FP3** and monomer, respectively, both with respect to Fc|Fc^+^, see ESI†). Thus, **FP3** is likely to be in an oxidized form upon physisorption onto the graphene electrodes, (in fact, we show that it is oxidized to the dicationic *N* − 2 **FP32+** state at *V*_g_ = 0, *vide infra*). We can therefore safely attribute the sequential tunneling regions that are observed in the experimental stability diagrams to the transitions between different charge states of **FP3** as electrons tunnel into and from the highest occupied orbitals (of the neutral species). The (closely spaced) HOMO/HOMO−1 orbitals of **FP3**, the two orbitals emptied as the molecule is oxidized from *N* to *N* − 4 charge states (**FP3** to **FP34+**), are shown in Fig. 2a. The orbitals, by inspection, appear as an in-phase and out-of-phase (or bonding/anti-bonding) combination of ‘site’ orbitals that are primarily based on the electron-rich pyrene anchors (Fig. 2b). Thus, linear combinations of the delocalized HOMO/HOMO−1 can be taken to transform them into a localized ‘left’ and ‘right’ site orbital, *ϕ*_L_ and *ϕ*_R_ (Fig. 2a). By making this transformation, from an eigenbasis to site basis, the many-body electronic structure of **FP3** in the five oxidation states from *N* → *N* − 4 can be modeled using a two-site extended Hubbard dimer model in which the left site couples only to the left electrode and *vice versa*, and the two sites are coupled to each other.

The Hamiltonian of the full system is given by:

1 *H* = *H*_E_ + *H*_V_ + *H*_HB_

where the left (L) and right (R) electrodes are fermionic reservoirs described by:2
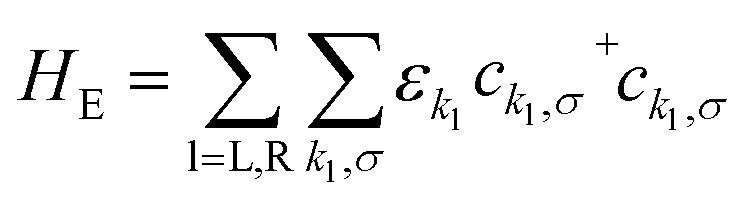
that are coupled to **FP3***via* the Hamiltonian:3
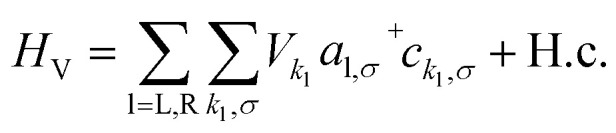


The extended Hubbard Hamiltonian that describes the many-body electronic structure of **FP3** is given by:4
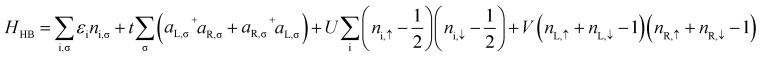
where *t*, *U* and *V* are the inter-site tunnel coupling, on-site repulsion and inter-site repulsion, respectively (Fig. 2b). *a*_i,σ_^+^ and *a*_i,σ_ are creation and annihilation operators for an electron of spin σ (= ↑ or ↓) in site i (= L or R). *n*_i,σ_ are the number operators, *n*_i,σ_ = *a*_i,σ_^+^*a*_i,σ_. Creation and annihilation operators for an electron of energy *ε*_*k*_l__ in the electrodes are given by *c*_*k*_l_,σ_^+^ and *c*_*k*_l_,σ_ and *V*_*k*_l__ is the coupling strength. We apply the wide-band approximation and take: *V*_*k*_l__ = *V*_l_. This is related to the molecule–electrode coupling by: *Γ*_l_ = 2π|*V*_l_|^2^*ρ*_l_ under the assumption that the density of states in the leads, *ρ*_l_, is constant.^30^

The energies of the molecular states, *ε*_i_ depend on the bias and gate voltages:

5 *ε*_i_ = *ε*_0_ − *α*_s_*V*_b_ − *α*_g_*V*_g_

where *α*_s_ and *α*_g_ are the coupling to the source and gate electrodes respectively.

For the two-site molecular system, which can accommodate up to four electrons, the eigenvectors of the Hubbard Hamiltonian are summarized in Table 1.^31^ The ‘vacuum’ state corresponds to both HOMO and HOMO−1 being empty (*N* − 4: **FP34+**); the neutral molecule (*N* state) is when the HOMO−1 and HOMO are both filled. Therefore, there is a single electronic state for *N* − 4 and *N* charge states. For each of *N* − 1 and *N* − 3 there are a pair of doubly (spin) degenerate states, separated in energy by 2|*t*|, denoted D^−^ and D^+^. Finally for *N* − 2 there exist 6 states: a 3-fold degenerate triplet T, and three singlet states, analogous to a 2-orbital-2-electron treatment.^32^ The nature of the singlet states is more complex than an open-shell/closed-shell description, as shown in Table 1.

**Table 1:** The 16 energy eigenvectors of the Hubbard Hamiltonian for charge states *N* − 4 to *N* and their designation, under the assumption of equal site energies. σ = (↑,↓), *Φ*_A_ = |↑↓,0〉, *Φ*_B_ = |0,↑↓〉, *Φ*_C_ = |↑,↓〉 and *Φ*_D_ = |↓,↑〉. The coefficients *c*_+_ and *c*_−_ depend on the values of *t*, *U*, and *V*, as described in the text

Charge state	Eigenstates of *H*_HB_	State (degeneracy)
*N* − 4	|0,0〉	*S*^*N*−4^ (1)
*N* − 3	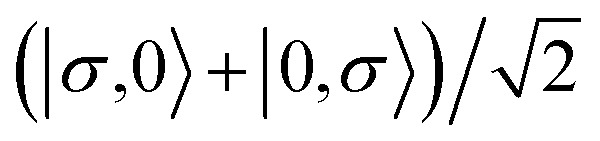	*D*_+,σ_^*N*−3^ (2)
	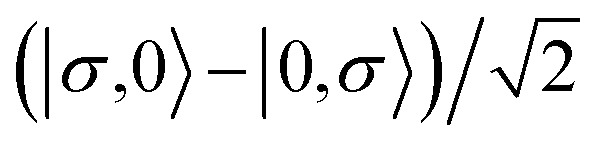	*D*_−,σ_^*N*−3^ (2)
*N* − 2	*c*_−_(*Φ*_A_ + *Φ*_B_) − *c*_+_(*Φ*_C_ − *Φ*_D_)	*S*_−_^*N*−2^, (1)
	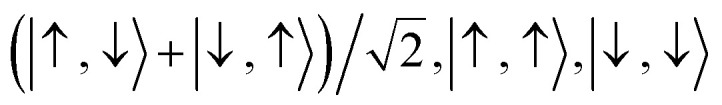	*T*_0_^*N*−2^, *T*_1_^*N*−2^*T*_−1_^*N*−2^ (3)
	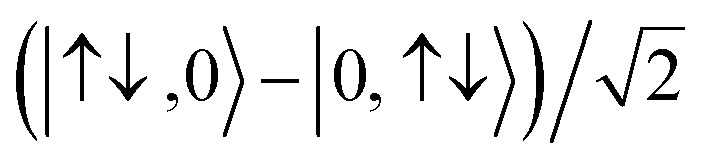	*S*_CS_^*N*−2^, (1)
	*c*_+_(*Φ*_A_ + *Φ*_B_) + *c*_−_(*Φ*_C_ + *Φ*_D_)	*S*_+_^*N*−2^, (1)
*N* − 1	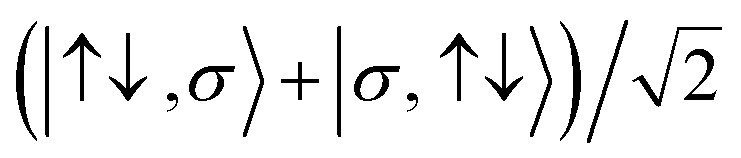	*D*_+,σ_^*N*−1^, (2)
	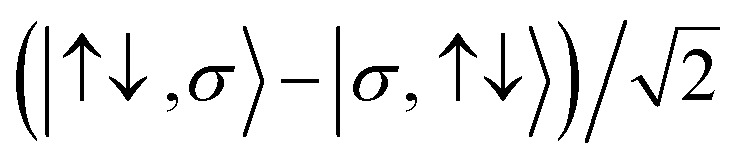	*D*_−,σ_^*N*−1^, (2)
*N*	|↑↓,↑↓〉	*S*^*N*^, (1)

The coefficients in Table 1, *c*_+_ and *c*_−_ are given by:6
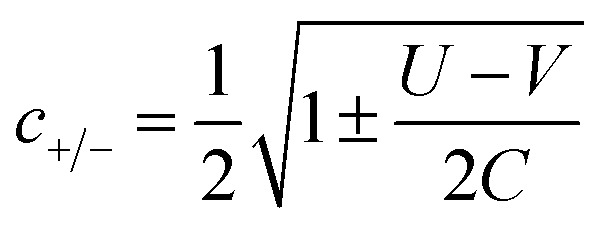
where *C* is given by:7
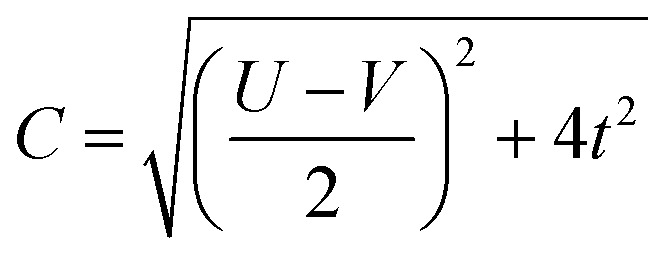
giving some of the *N* − 2 singlet states a mix of open-shell/closed shell character that depends on the size of the on-site and inter-site repulsion, and inter-site hopping.

For transport through a number of electronic states, the current through the molecular junction can be compactly calculated using a rate-equation-type framework by first constructing the (in this case 16 × 16) rate-equation matrix, ***W***.^31,33^ Taking the steady state approximation, d***P***/d*t* = ***WP*** = 0, the stationary occupation probabilities of the 16 electronic states ***P***_0_ are the null space of ***W***, normalized such that the elements of ***P***_0_ are non-negative and sum to 1.^33^ The total current is then calculated by considering the tunneling processes at either electrode. The elements of ***W*** are comprised of the electron-transfer rates between states *j* and *k*, *γ*_l_^*j*→*k*^.

The electron-transfer rates are given by:8
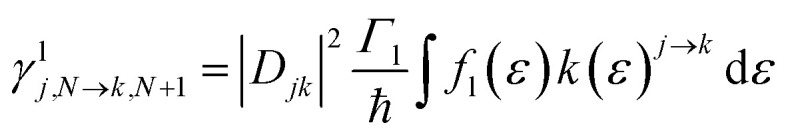
9
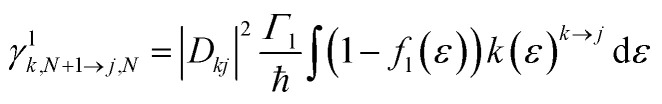
for reduction and oxidation respectively at electrode l (=L/R for left/right electrode). *Γ*_l_ is the molecule–electrode coupling, *f*_l_(*ε*) is the Fermi–Dirac distribution of electron energies in electrode l. The electron-transfer rate constants, *k*(*ε*)^*j*→*k*^, are Dirac delta functions centered at the chemical potential of the transition from j to k. As we will show later, these functions can be replaced with energy-dependent rate constants that also account for electron–vibrational coupling accompanying electron-transfer. *D*_*jk*_ is the overlap integral 〈*ϕ*_k_|*a*_i,σ_^+^|*ϕ*_j_〉, also known as the Dyson orbital coefficient. Inclusion of these coefficients precludes the need to include statistical factors based on degeneracies into the rate-equation matrix. Furthermore, they automatically encode the selection rules for electron transfer: Δ*S* = ±1/2 and Δ*m*_S_ = ±1/2. For instance, the transitions from the *D*_+,↑_^*N*−3^ state to the *T*_0_^*N*−2^, *T*_1_^*N*−2^*T*_−1_^*N*−2^ states result in Dyson coefficients of 1/2, 
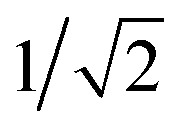
 and 0, respectively. That is, if the molecule is in a *D*_+,↑_^*N*−3^ state, it has a single, spin-up electron (*m*_S_ = ½), and so one additional electron cannot hop in to create the state *T*_1,↓_^*N*−2^ which has two spin-down electrons (*m*_S_ = −1) so 
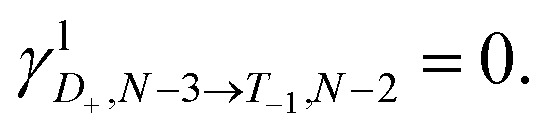


### Experimental charge stability diagrams

**Fig. 3:**
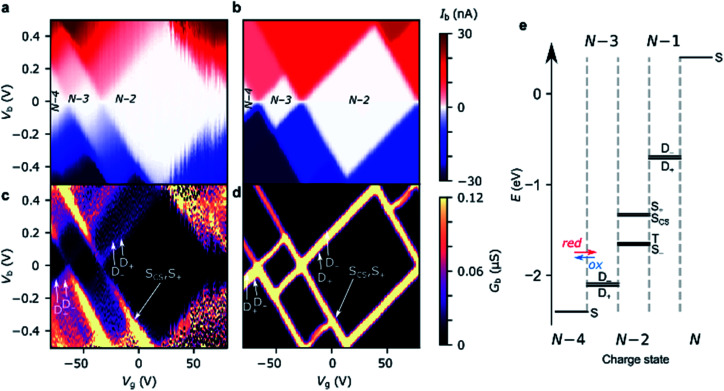
Experimental stability diagrams of device **A**, (a) current and (c) derived conductance, measured at 77 K; the Coulomb diamonds are assigned with their charge states. (b) and (d) are current and conductance stability diagrams, respectively, calculated using the Hubbard model, with parameters *U* = 0.50 eV, *V* = 0.14 eV and *t* = −0.01 eV. The couplings to the source and gate electrodes are *α*_S_ = 0.37, and *α*_G_ = 4.8 × 10–3, taken from the experimental data. The molecule–electrode couplings are taken from the *IV* fit in Fig. 4a. (e) The energy eigenvalues of the 16 Fock states of **FP3** involved in electron transfer calculated for device A using the Hubbard model at *V*_g_ = −80 V, the lowest experimental gate voltage. The potentials experienced by the molecule are much lower than those applied experimentally due to screening by the 300 nm SiO_2_ gate dielectric, this is captured by the small value of *α*_G_.

Stability diagrams of **FP3** devices are given in Fig. 3 (device **A**) and ESI† (device **B** and **C**). Multiple sequential tunneling regions are observed, as expected, reflecting the redox activity of the edge-fused trimer with respect to the monomer.^7^ A common feature of the charge stability diagrams of devices **A–C** is the presence of a larger Coulomb diamond centered around *V*_g_ = 0, flanked by smaller diamonds with addition energies ranging from 0.14–0.30 eV. The addition energy, *E*_add_, is the energy required to add an extra electron to a molecule in the device, and can be read directly from a stability diagram as the width of the corresponding Coulomb diamond (scaled by the gate coupling, *α*_G_).^21^ The energy eigenvalues of the Hubbard Hamiltonian can be translated into analytical expressions for addition energies of the *N* − 1, *N* − 2 and *N* − 3 charge states by taking the energy of the ground state of each charge state. In the limit of *U*, *V* ≫ *t*, the addition energies are *V*–*t* for the odd diamonds *N* − 1 and *N* − 3, and *U* for *N* − 2. By considering the experimental addition energies of device **A** in the extended Hubbard framework, we are able to determine the electron–electron repulsion terms *U* ≈ 0.5 eV and *V* ≈ 0.14 eV; from the rectification behavior and DFT calculations (discussed below), we infer below that *U*, *V* ≫ *t*. We know that |*t*| must be non-zero (for transport to occur) and negative (due to the spatial properties of the orbitals, see Fig. 2). The value of *t* = −0.01 eV is in agreement with our later experimental observations. The calculated stability diagram, using coupling to the electrodes determined from the slopes of the experimental Coulomb diamonds, is shown in Fig. 3b. Not only does the extended Hubbard model predict the positions of the edges in the stability diagram, it is also simultaneously consistent with other experimental observations, *i.e.* gate-dependent rectification ratios and high-bias excited states, as outlined in the following paragraphs.

**Fig. 4:**
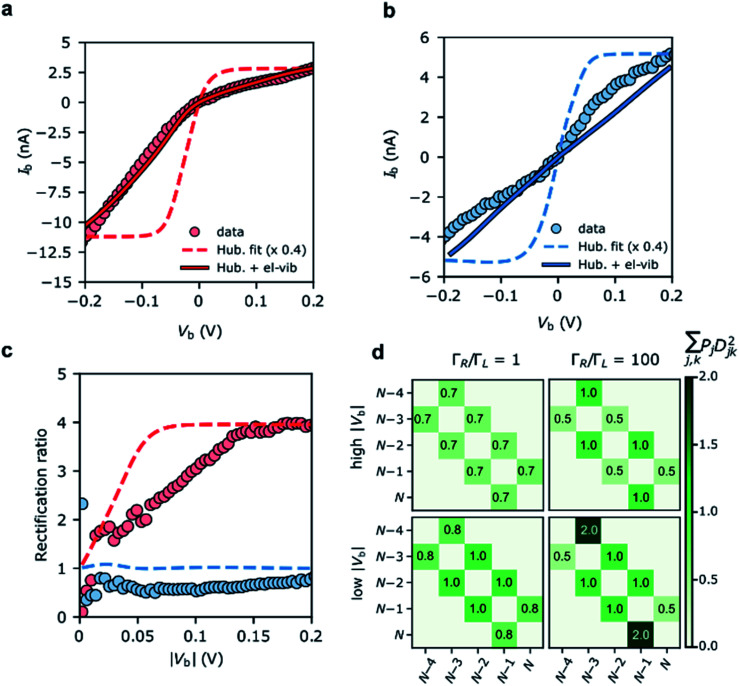
Device **A***IV* traces on (a) the *N* − 4/*N* − 3 resonance (*V*_g_ = −66 V), and (b) the *N* − 3/*N* − 2 resonance (*V*_g_ = −34 V). The experimental data are plotted alongside *IV* traces taken from the Hubbard stability diagrams in Fig. 3, and the Hubbard model plus electron–vibration coupling included in the electron-transfer rates. Electron–vibration fitting parameters for *N* − 4/*N* − 3: *Γ*_L_ = 41 μeV, *Γ*_R_ = 14 meV, *λ*_0_ = 70 meV; for *N* − 3/*N* − 2, *λ*_0_ = 120 meV. (c) The rectification behaviour, *I*_b_(−*V*_b_):*I*_b_(+*V*_b_), of the *N* − 4/*N* − 3 (red) and *N* − 3/*N* − 2 (blue) transitions are given for the experimental values (circles) and the Hubbard model (dashed lines). The measurements were taken at a device temperature of 77 K. (d) The emergence of the rectification behaviour of the Hubbard model under asymmetric molecule–electrode coupling. Each element (*j*,*k*) represents the sum of the electron transfer rates from charge state *j* to *k*. ‘High’ |*V*_b_| means above 2|*t*|/*α*_S_, when both *N* − 3 doublets are within the bias window. An improved fit to *N* − 3/*N* − 2 (maintaining the rectification ratio) can be achieved by going beyond the wide-band gap approximation, see ESI.†

For transport through a single spin-degenerate level the rectification ratio varies between 1/2 and 2 depending on the asymmetry of the molecule–electrode coupling.^29^ The *N* − 4/*N* − 3 transition (at *V*_g_ = −66 V in Fig. 4a) exhibits a rectification ratio of nearly exactly 4 (see Fig. 4c), a clear deviation from what is observed for a single spin-degenerate level. That rectification ratio is also present in the corresponding *IV* traces of device **B** and **C**.

Due to the asymmetry in the molecule–electrode couplings (present in all devices considered here) the rectification ratio of the resonant *IV* trace can be used to directly infer the number of electronic states within the bias window.^34,35^ For device **A**, *Γ*_R_ ≫ *Γ*_L_, and so a ratio *I*_b_(−*V*_b_) : *I*_b_(+*V*_b_) of 4 : 1 indicates the rate of tunneling onto the molecule is four times greater than tunneling off. From this we can infer that there are four available states for reduction of the *N* − 4 state, but only a single state that can be accessed from the oxidation of the *N* − 3 state. The observed rectification in the experimental data is inherently captured by the Hubbard model (Fig. 4a and c). The energy spacing between the *D*_+_^*N*−3^ and *D*_−_^*N*−3^ levels is only 2|*t*| = 20 meV, and therefore both levels are found within the bias window at above roughly 50 mV. Once both the low-lying doublets *D*_+_^*N*−3^, and *D*_−_^*N*−3^ are within the bias window, there are four *N* − 3 states that *S*^*N*−4^ can be reduced to when an electron hops onto the **FP34+**, but each state can only be oxidized back to *S*^*N*−4^, see Fig. 3e. At 77 K, the transition from 1 : 2 to 1 : 4 rectification ratios as *D*_−_^*N*−3^ enters the bias window is significantly broadened due to the lifetime-broadening, the Fermi functions in the leads, but mainly due to the energy-dependence of the hopping rates that results from electron–vibrational coupling (not accounted for in the Hubbard model). Therefore, the excited state transition *S*^*N*−4^ ↔ *D*_−_^*N*−3^ is not visible as a separate parallel line intersecting the *N* − 3 Coulomb diamond, and, this observation is consistent with an estimated value of *t* that is of the same order as *k*_B_*T* (8 meV).

For the *N* − 3/*N* − 2 transition (at *V*_g_ = −34 V), the experimental rectification ratios are around 1 (see Fig. 4b and c). This is again in agreement with the Hubbard model. From Fig. 3e, this ratio arises because charge transport at higher bias occurs between four doublets of the *N* − 3 charge state and the ground-state singlet and the low-lying triplet of the *N* − 2 charge state (the singlet–triplet gap is only approximately ∼1 meV for the values of parameters used in the Hubbard model). The remaining excited singlet states are visible in Fig. 3 as the excited state line at higher bias. The situation becomes slightly more nuanced as the probability of each transition is scaled by a relevant Dyson orbital coefficient. The rectification behavior can be understood for the full set of transitions from Fig. 4d, the off-diagonal elements connecting two charge states represent the rate of transfer between those two states on resonance. By inspection of the upper left corner we can see that the rate of *N* − 4 → *N* − 3 is 2.0 whereas it is 0.5 for *N* − 3 → *N* − 4, giving a ratio of *I*_b_(−*V*_b_) : *I*_b_(+*V*_b_) of 4 : 1. For *N* − 3/*N* − 2 it is 1.0 for either direction. These values also reflect the relative magnitudes of current expected between the *N* − 4/*N* − 3 and *N* − 3/*N* − 2 transitions for a Hubbard dimer, as is observed experimentally.

### Electron–vibrational coupling

Due to their relatively small size, molecular systems undergo significant geometric changes upon charging when compared to lithographically defined structures, and as such vibration coupling to sequential electron transport is significant for these systems. **FP3** has 3*N*_atom_ − 6 = 3345 vibrational normal modes that span the energies between a few meV for out-of-plane bending motions, through several hundreds of meV for C–C bond stretches and up to 400 meV for C–H stretches, as is typical of a large π-conjugated molecule. Electron–vibration coupling to these modes causes low-bias suppression of tunneling current, and by omitting them, *IV* traces calculated from the Hubbard model significantly overestimate the current at low bias, as can be seen in Fig. 4a and b. The absence of electron–vibration coupling in the Hubbard model also accounts for the lack of asymmetry in the sequential tunneling regions with respect to gate voltage that is visible in the experimental stability diagrams.^29^ In order to reproduce absolute values of the current and therefore reinforce the fact that the two-site Hubbard model is applicable, we incorporate electron–vibration coupling into the electron-transfer rate constants, *k*^*i*→*j*^, by replacing the Dirac delta functions centered on the chemical potential of the transition from *i* to *j*.

The method we use follows previous work,^7,30^ and is described in more detail in the ESI.† In short, a spectral density is constructed that accounts for contributions to the rates of electron transfer from the inner sphere (*i.e.* distortion of the molecule along normal modes of vibration upon charging) and from the outer sphere (*i.e.* distortion of the local molecular environment, predominantly the substrate). For the *N* − 4/*N* − 3 transition, the electron-transfer rates for reduction: *k*^*S*→*D*+^ and *k*^*S*→*D*−^ (and similarly for oxidation, *k*^*D*+→*S*^ and *k*^*D*−→*S*^) are assumed to be the same except for the offset in energy by spacing between the doublets, |2*t*|. The experimental *N* − 4/*N* − 3 *IV* traces are then fitted using three parameters, *λ*_0_, *Γ*_S_, and *Γ*_D_, to reproduce the experimental data (Fig. 4a). The fits to the *N* − 4/*N* − 3 transitions for device **B** and **C** are given in the ESI.†

The *N* − 3/*N* − 2 resonant *IV* curve can be fitted following the same method. The geometry of the *N* − 2 state (**FP32+**) is optimized in the singlet or triplet ground state to calculate *k*^*D*+→*S*−^ and *k*^*D*+→*T*^. As with the *N* − 4/*N* − 3 transition, we assume the geometry of the doublets, *D*_+_^*N*−3^ and *D*_−_^*N*−3^ are the same. The molecule–electrode couplings from the *N* − 4/*N* − 3 fit are used and therefore *λ*_0_ is the only free parameter. Fig. 4 shows the inclusion of electron–vibration coupling converts the Hubbard *IV*s, which give the required rectification ratios, into good fits to the experimental data.

### DFT calculations

**Fig. 5:**
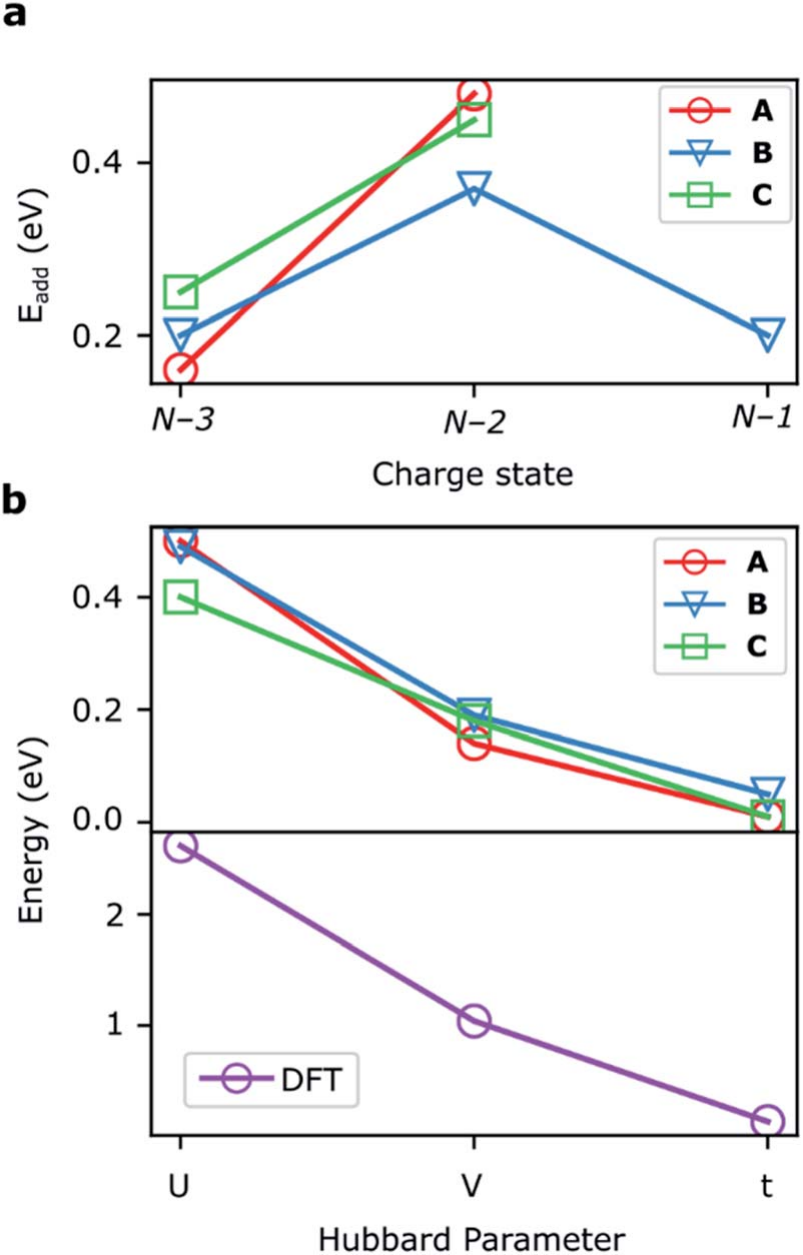
(a) Addition energies from experimental stability diagrams for devices **A–C**. (b) The Hubbard parameters for the devices that reproduce these addition energies (upper panel) along with DFT calculations of these values (lower panel).

The addition energies for **A**, **B**, and **C** are given in Fig. 5a; the devices follow the same trend with only slight variations in the values of *U*, *V*, and *t* needed to be selected for each device. The values of these parameters extracted from the experimental charge stability diagrams, which are seemingly intrinsic to the molecular structure, can be compared to those calculated using DFT. Electron–electron repulsion terms are the Coulomb integrals: *U* = 〈*ϕ*_L_*ϕ*_L_|1/(4π*ε*_0_*ε*_r_*r*_12_)|*ϕ*_L_*ϕ*_L_〉 and *V* = 〈*ϕ*_L_*ϕ*_L_|1/(4π*ε*_0_*ε*_r_*r*_12_)|*ϕ*_R_*ϕ*_R_〉. For optimized gas-phase geometries (*ε*_r_ = 1) the values are *U*_DFT_ = 2.62 eV and *V*_DFT_ = 1.0 eV (Fig. 5b). The kinetic term, *t*, is obtained from DFT calculations as half the difference between the HOMO/HOMO−1 (see Fig. 2a), which yields *t*_DFT_ = −0.13 eV. Values very similar to those obtained experimentally can be obtained by introducing an effective *ε*_r_ that accounts for the dielectric environment. If we set *ε*_r_ to 5.5 (a value comparable other π-conjugated organic molecules^36^) we obtain 
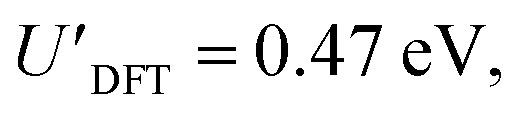
 and 
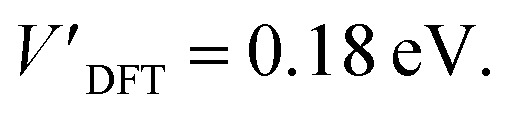
 This effective dielectric constant can account for intramolecular charge screening as well as polarization of the oxide substrate and the graphene electrodes.^37^ The kinetic energy term *t* does not scale linearly with *ε*_r_, however it can still be altered by the electrostatic influence of the substrate and geometric distortions. The binding energies of the anchor groups to the graphene are estimated to be several eV,^38^ and therefore, for each molecular junction, where the exact atomic structure of the electrodes are unknown, the molecule can readily adopt a unique conformation to maximize binding. As an example of one of many possible low-energy distortions of the molecular geometry, *t* has a strong dependence on the dihedral angle between the porphyrin trimer and the anchor groups (see ESI†). In addition, this coordinate modifies the **FP32+** singlet–triplet energy spacing (which are calculated using the Hubbard model to be 1.2 meV, 1.1 meV, and 30 meV for devices **A**, **B** and **C**, respectively). Therefore, the device-to-device variation observed is most likely due to both differences in molecular conformation, and the unique local dielectric environment for each device. This further highlights the requirement that if highly reproducible single-molecule device characteristics are desired, precise control over the molecular environment is necessary.

## Conclusions

In conclusion, we report on the sequential transport behavior of an edge-fused porphyrin trimer in a single-molecule junction at 77 K. The large, conjugated molecular structure and weak molecule–electrode coupling lead to multiple sequential tunneling regions that are experimentally accessible. This allows us to study the many-body electronic structure of this system in various charge states, and understand the resulting transport properties and energy scales of the junction. As the Coulomb interactions dominate the energetics of the system (*i.e. U*, *V* ≫ *Γ*, *k*_B_*T*) and considering the spatial distribution of the orbitals involved in transport, the **FP3**-graphene molecular junction can be modeled as a two-site Hubbard dimer. Due to the redox activity of the molecule we infer observables resulting from electron correlations, such as the singlet–triplet splitting in the *N* − 2 state, that may otherwise be inaccessible experimentally. Uniquely amongst related two-site molecules,^39,40^ the molecule is fully conjugated between the two sites, apparently negating any voltage drop across the molecule. The Hubbard framework reproduces key features of the experimental stability diagrams that pertain to many-body electron–electron interactions, *i.e.* the addition energies, rectification ratios (which indicate the presence of excited states involved in electron transfer), and high bias excited states. A quantitative reproduction of the experimental *IV* curves requires integrating electron–vibrational interactions into the Hubbard model.

These experiments may guide future explorations of the role of electron correlations in charge transport through extended aromatic systems such as longer porphyrin tapes or graphene nanoribbons.^41^ The tunneling current through the molecule depends on the interplay between *t* and *Γ* (the molecule–electrode coupling). In our **FP3** devices, it is always one of the molecule–electrode couplings (either *Γ*_S_ or *Γ*_D_) that limits the current, as one of them is always much smaller than *t*. This parameter *t*, which is half the HOMO/HOMO−1 energy gap, quantifies the strength of coupling between the two sites. In longer fully-conjugated porphyrin tapes with the same anchoring groups, we expect the coupling between the sites, *t*, to remain strong with, so that the molecules conductance will still be limited by the weakest molecule–electrode coupling, while we expect *V* (intersite repulsion) to be much smaller due to the increased distance between the sites. The addition energies for the odd charge states are *V*–*t*, so we predict very small Coulomb diamonds for odd charge states, but relatively similar ones for the even charge states, as *U* is expected to be independent of the length of the tape. Furthermore as *t* can be controlled by chemical modification of the porphyrin–porphyrin connection within oligomer,^23^ oligomers with weak intramolecular tunnel coupling can be synthesized to investigate phenomena such as rectification due to Pauli spin blockade^31,42^ in single-molecule devices.

The study of strongly correlated electrons within extended molecular structures is not only relevant to charge transport. For example, the many-body electronic structure of related oligomeric systems could be probed by spectroelectrochemistry,^43^ and the ring currents in oxidized oligomers of different connectivity, measured in NMR spectroscopy,^44,45^ could also be rationalized using similar frameworks.^46^

## Data availability

The data is available within the main text and associated ESI files.

## Author contributions

JOT: conceptualization, data curation, formal analysis, investigation, methodology, validation, visualization, writing – original draft. JKS: formal analysis, methodology, writing – original draft. BL: conceptualization, investigation, writing – review & editing. XB: investigation, visualization, writing – review & editing. CE: investigation, writing – review & editing. JLS: investigation, writing – review & editing. ST: investigation, writing – review & editing. JB: resources, writing – review & editing. GCS: supervision, writing – review & editing. GADB: conceptualization, funding acquisition, resources, writing – review & editing. HLA: conceptualization, funding acquisition, resources, supervision, writing – review & editing. JAM: conceptualization, methodology, funding acquisition, resources, supervision, writing – original draft.

## Conflicts of interest

There are no conflicts to declare.

## Supplementary Material

SC-012-D1SC03050G-s001
