# Supplementary material for: Charge transport through extended molecular wires with strongly correlated electrons
Source: Chem Sci. 2021 Jul 26;12(33):11121–9. doi: 10.1039/d1sc03050g (PMC8386642; doi:10.1039/d1sc03050g)
Supplement: SC-012-D1SC03050G-s001 [file SC-012-D1SC03050G-s001.pdf]

Electronic Supplementary Material (ESI) for Chemical Science.
This journal is © The Royal Society of Chemistry 2021

# Electronic Supplementary Information: Charge transport through extended molecular wires with strongly correlated electrons

James O. Thomas,[ab]* Jakub K. Sowa,[c,d] Bart Limburg,[ab] Xinya Bian,[a] Charalambos Evangeli,[a] Jacob L. Swett,[a] Sumit Tewari,[a] Jonathan Baugh,[e] George C. Schatz,[c] G. Andrew D. Briggs,[a] Harry L. Anderson[b] and Jan Mol[f]

[a] Department of Materials, University of Oxford, Parks Road, Oxford, OX1 3PH, UK

[b] Department of Chemistry, University of Oxford, Chemistry Research Laboratory, Oxford OX1 3TA, UK

[c] Department of Chemistry, Northwestern University, Evanston, Illinois 60208, USA

[d] Department of Chemistry, Rice University, Houston, TX, USA

[e] Institute for Quantum Computing, University of Waterloo, Waterloo, ON, N2L 3G1, Canada

[f] School of Physics and Astronomy, Queen Mary University of London, London, E1 4NS, UK

## Contents

Synthetic Procedure and Characterisation ........ 2
Device Fabrication........ 5
Electrical Measurements ........ 5
Supporting Devices ........ 7
Device A - Additional *IV* Fit........ 9
Estimation of t from IV traces. ........ 11
Electron-Vibration Coupling........ 12
Calculation of Electron-Phonon Coupling Constants ........ 13
DFT calculation of *N–2* singlet-triplet energy spacing and *t* ........ 16
ESI References........ 17

## Synthetic Procedure and Characterisation

**FP3** was synthesized by Sonogashira coupling, as shown in Scheme 1. The **$Br_2$FP3** and **TDP** building blocks were made according to literature procedures.[1, 2] All reagents were obtained from commercial sources and used as received without further purification. Toluene and DIPA were taken from a solvent drying system (MBraun MB-SPS-5-Bench Top) under nitrogen. Petroleum ether (PE) with a 40–60 °C boiling point range was used. Column chromatography was carried out using Merck Geduran silica gel 60 under $N_2$ pressure. TLC was carried out on Merck silica gel 60 F254 Al plates. MALDI-TOF-MS was carried out in positive reflectron mode using a Bruker MALDI microflex instrument with dithranol as a matrix. NMR spectroscopy measurements were recorded using a Bruker AVII400 instrument. All peaks were referenced to the residual solvent peak.

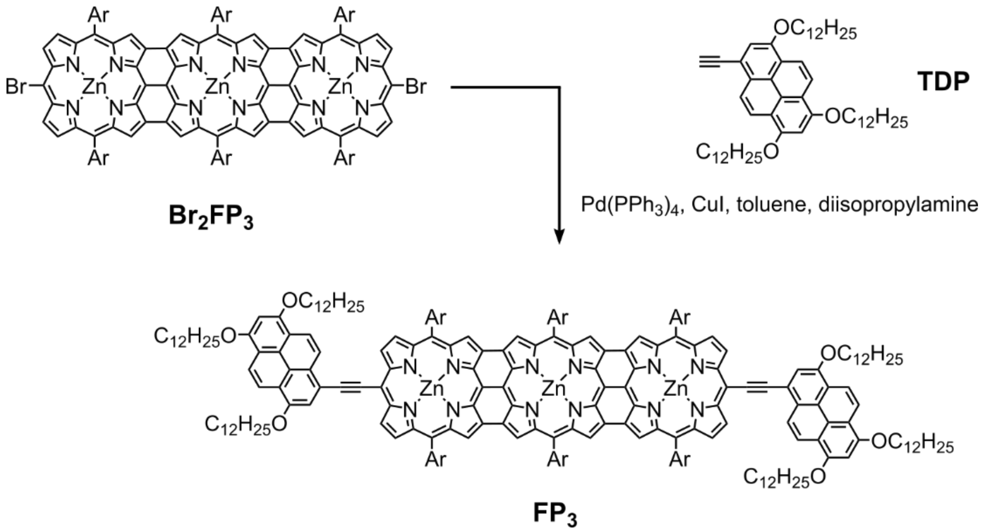


**Scheme 1.** Synthesis of fused porphyrin trimer with tridodecyloxypyrene anchoring groups. Ar = 3,5-bis(trihexylsilyl)phenyl.

<u>FP3</u>

**$Br_2$FP3** (2.35 mg, 0.46 µmol) and **TDP** (1.79 mg, 2.3 µmol) were dissolved in toluene (0.8 mL) and DIPA (0.2 mL). The solution was degassed by freeze-pump-thaw three times before [Pd(PPh3)4] (0.1 mg, 0.08 µmol) and CuI (0.02 mg, 0.1 µmol) were added under a flow of argon. After another freeze-pump-thaw cycle, the mixture was heated to 50 °C for 3 hours. The solvents were removed and the product purified by chromatography (SiO2, PE:DCM 4:1). Yield: 1.80 mg, 60%. m/z (MALDI-TOF, dithranol): 6512.6963 ([M]+ calcd. 6511.17).

$^1$H NMR (400 MHz, $CDCl_3$) $\delta_H$ / ppm: 8.74 (d, J = 4.4 Hz, 4H), 8.50 (d, J = 9.2 Hz, 2H), 8.31 (d, J = 9.1 Hz, 2H), 8.26 (d, J = 9.2 Hz, 2H), 8.18 (d, J = 9.3 Hz, 2H), 7.79 – 7.73 (m, 14H), 7.66 (s, 2H), 7.57 – 7.48 (m, 8H), 7.07 – 7.03 (m, 6H), 6.31 (s, 4H), 4.35 – 4.17 (m, 12H), 2.02 – 1.85 (m, 12H), 1.64 – 1.49 (m, 12H), 1.42 – 1.04 (m, 384H), 0.86 – 0.63 (m, 206H).

MALDI-TOF m/z: 6512.6963 ([M]+ calcd. 6511.17).

UV/vis/NIR ($CHCl_3$, 298 K) $\lambda_{max}$ / nm (log ε): 432 (4.95), 731 (5.07), 1533 (4.91)

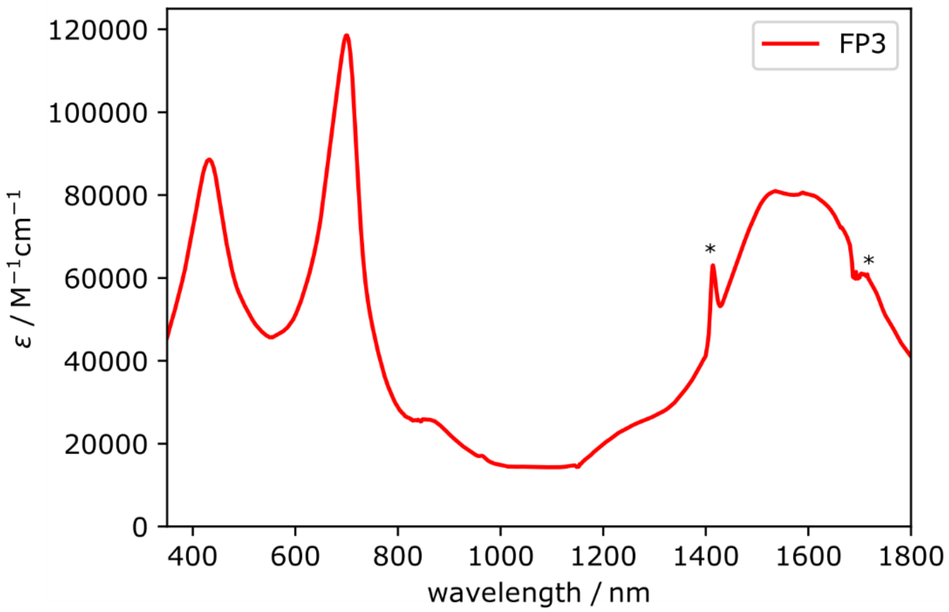


Figure S 1/ UV/vis/NIR spectrum of **FP3** in $CHCl_3$ at 298 K. Peaks indicated by the asterisks are due to errors in subtraction of the solvent background.

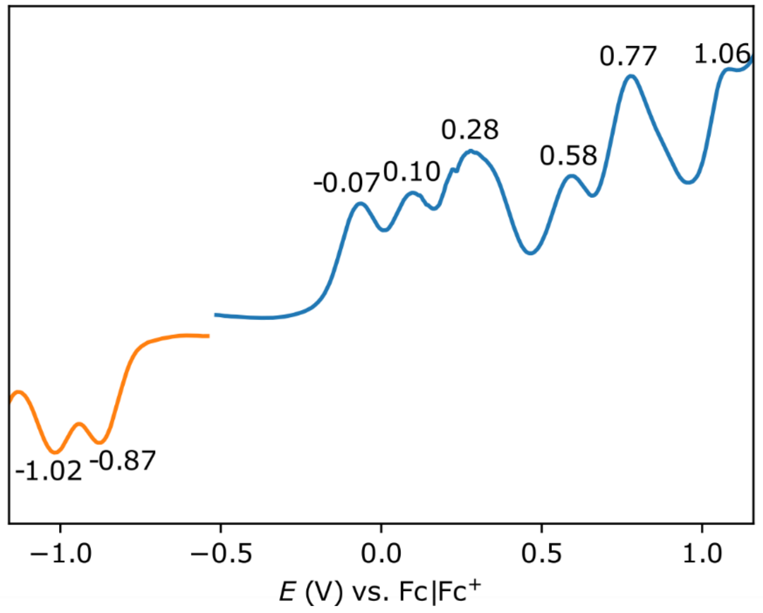


Figure S 2. Square wave voltammogram of **FP3**, showing a HOMO-LUMO gap of 0.80 eV. The spectrum was recorded in a 0.1 M tetrabutylammonium hexafluorophosphate DCM solution as the electrolyte. Glassy carbon, platinum wire, and Ag|$AgNO_3$ (10 mM) were used as the working, counter and reference electrodes respectively. Ferrocene was added at the end of the spectrum and the peaks reported are reference to the Fc+|Fc peak.

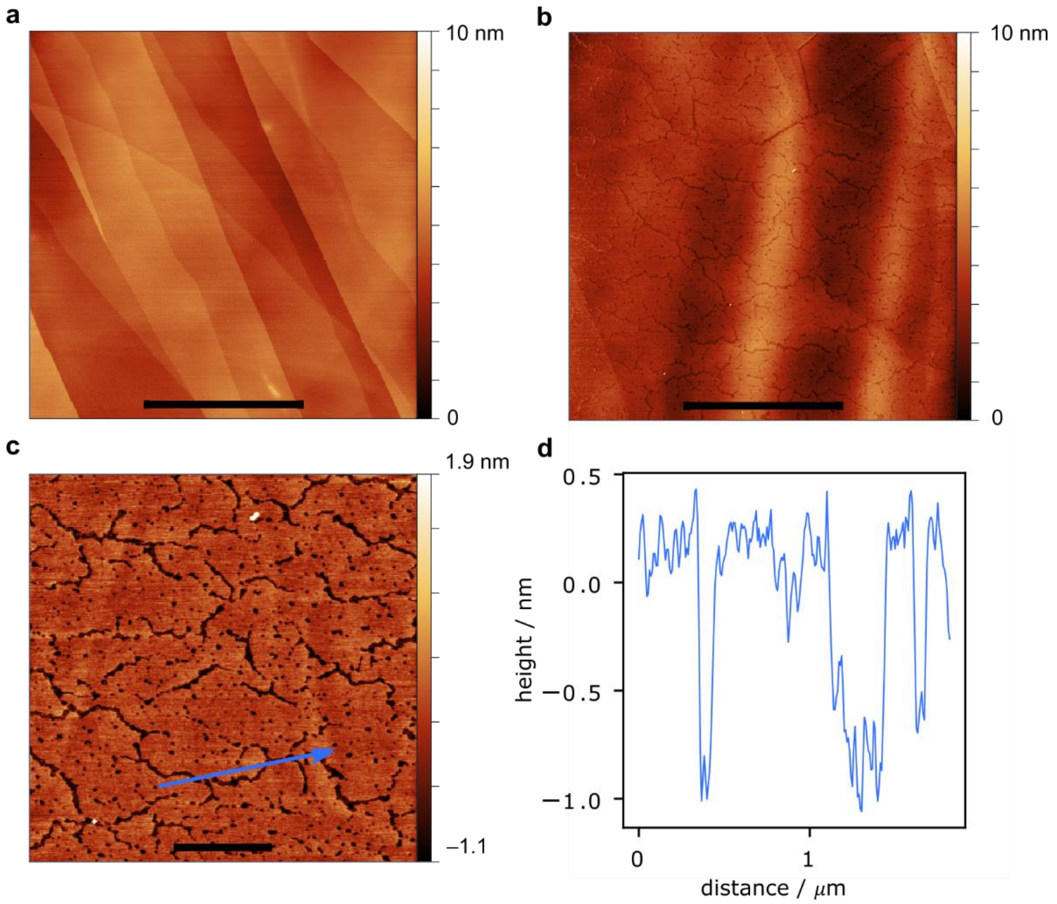


Figure S 3. AFM images recorded of HOPG before (**a**) and after (**b**) deposition of 12 µL of 2 µM **FP3** solution in toluene, corresponding to a sub-monolayer surface coverage. No evidence of aggregation is observed and the molecules form a complete monolayer. Scale bars: (a), 4 µm, (b) 4 µm, (c) 1 µm.

## Device Fabrication

Device fabrication followed previously reported procedures.[2, 3] Devices were fabricated on *n*-doped silicon wafers with 300 nm of thermally grown $SiO_2$. For device **A** and device **B** the underlying doped silicon was used as a global gate for all devices on each chip. For device **C** a local gate electrode was patterned. The local gates were fabricated by optical lithography and e-beam evaporation of titanium (10 nm) and gold (30 nm). A dielectric layer of $HfO_2$ (10 nm) was subsequently deposited by atomic layer deposition. Source and drain contact pads were patterned onto the $SiO_2$ (device **A** and **B**) or $HfO_2$ (device **C**) by optical lithography and e-beam evaporation of titanium and gold (10 nm / 60 nm).

CVD-grown monolayer graphene was transferred onto the devices by Graphenea. The graphene was patterned into bow-tie shapes with a width of approximately 100 nm at the narrowest point. First the devices were spin-coated with the negative tone resist ma-N 2403 and patterned using e-beam lithography with a dose of 120 $\mu C\ cm^{-2}$ and an accelerating voltage of 50 kV. The pattern was developed with ma-D 525 to remove the unexposed resist, and unprotected regions of graphene were etched by $O_2$ plasma. The developed resist was removed with an NMP-based remover REM660 to give the bowtie-shaped graphene. Finally, the patterned graphene was formed into a nanometer-spaced graphene tunnel junctions, graphene nano-gaps, by feedback-controlled electroburning[4, 5] with a threshold resistance of 600 MΩ. The IV curves after electroburning were fitted with the Simmons model to estimate the spacing between the graphene source and drain electrodes to be around 1.5 nm.

The gate-dependence of the source-drain current was measured at room temperature before deposition of the molecular solution on an automated probe station. The molecules were deposited onto the graphene nano-gaps from a 2 µM toluene solution, and the devices were measured again.

In total 950 devices were fabricated by feedback-controlled electroburning, and were measured before and after **FP3** deposition at room temperature. 101 devices were wire-bonded and cooled down to either 77 K or 4 K. 37 of these devices broke during wire-bonding or cool-down. 11 of the remaining 64 devices showed no sign of SET/Coulomb blockade, and 29 had gate coupling too weak or conductance too low to allow for further investigation. 10 showed a similar pattern of addition energies. 3 (device A, device B and device C) of these displayed N–4/N–3 transitions within the experimental window, and had sufficiently clean stability diagrams prior to molecular deposition to warrant modelling with the Hubbard framework.

## Electrical Measurements

Device **A** and device **B** were wire-bonded into a chip carrier and measured in a dip-stick setup at 77 K. The dip-stick was evacuated and immersed in a dewar of liquid nitrogen. A HP33120A function generator was used to apply the source-drain voltage. The gate voltage was applied by a Keithley 2450 SourceMeter. A Stanford Research Systems SR570 low-noise current amplifier was used to measure the source-drain current, and the data collected by a National Instruments BNC-2090A DAQ. Device **C** was measured in an Oxford Instruments 4K Pucktester. An Adwin Gold II data acquisition system was used to apply the source-drain and gate voltages. An SR570 was used to measure the current which was collected by the Adwin Gold II.

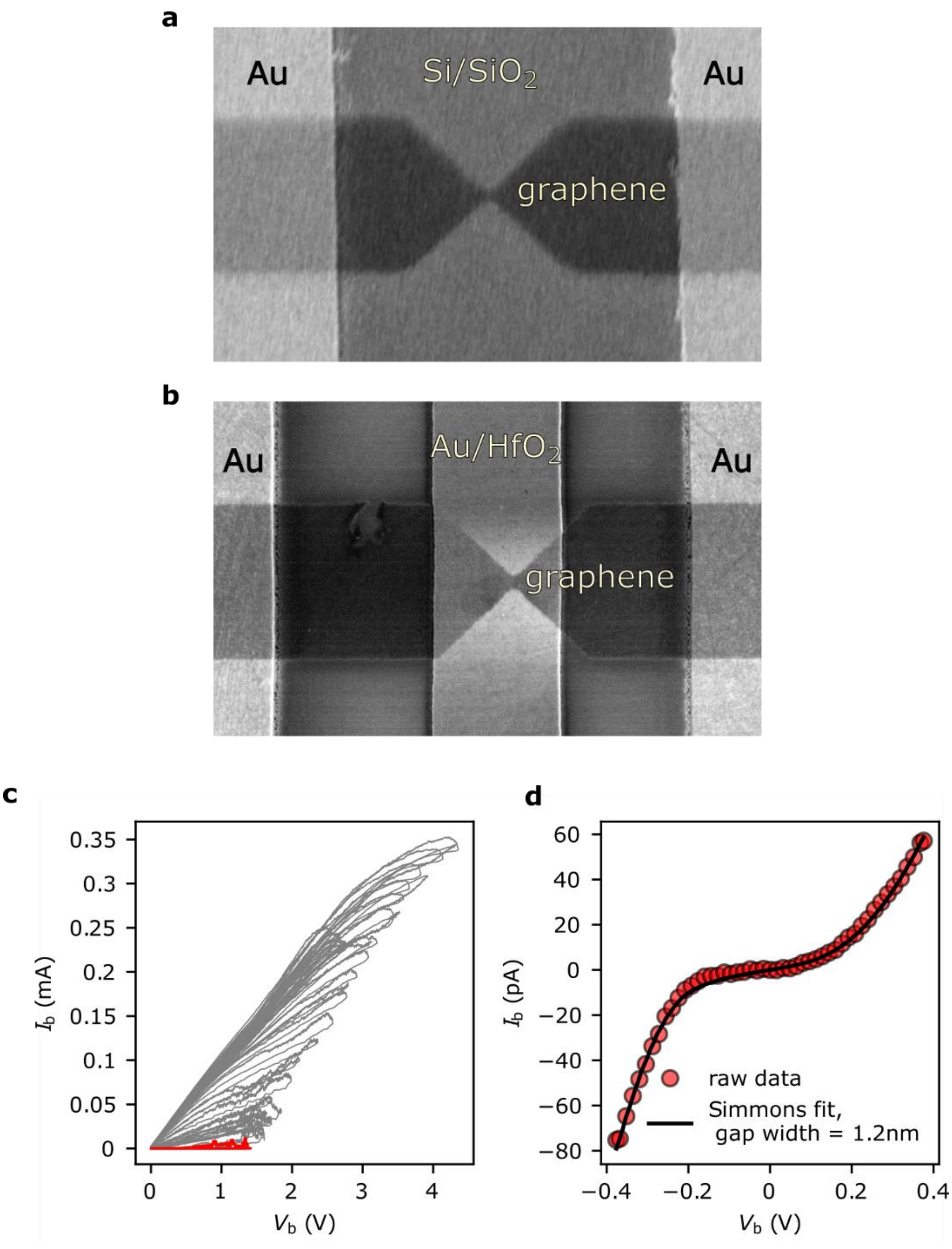


Figure S 4. SEM images of devices with a global Si/$SiO_2$ gate (**a**), and a local Au/$HfO_2$ gate (**b**). The feedback controlled electroburning curves, to form a graphene tunnel junction, for device **A** are shown in (**c**), the last trace is red. (**d**) The *IV* trace of device **A** directly after electroburning the graphene bowtie to a graphene tunnel junction. The fit to the Simmons gives a gap width of 1.2 nm.

## Supporting Devices

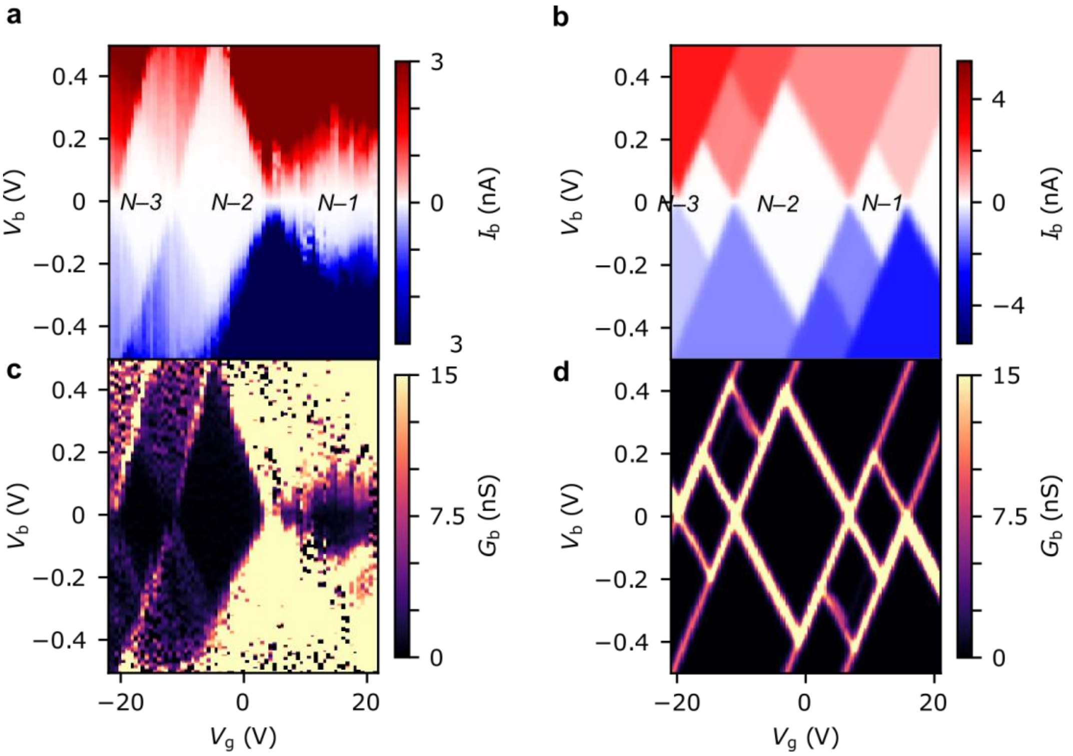


Figure S 5 Device **B**, measured at 77 K, experimental current (**a**) and conductance (**c**) stability diagrams. (**b**) and (**d**) are current and conductance stability diagrams calculated from the extended Hubbard model with parameters: *U* = 0.40, *V* = 0.18 and *t* = –0.01 eV. The values of *t,* $\Gamma_S$ and $\Gamma_D$ were taken from the fit to the *N–4/N–3* IV trace in Figure S6 . $\alpha_s$ and $\alpha_g$ were calculated from the gradients of the Coulomb diamond edges to be 0.57 and 0.022 respectively.

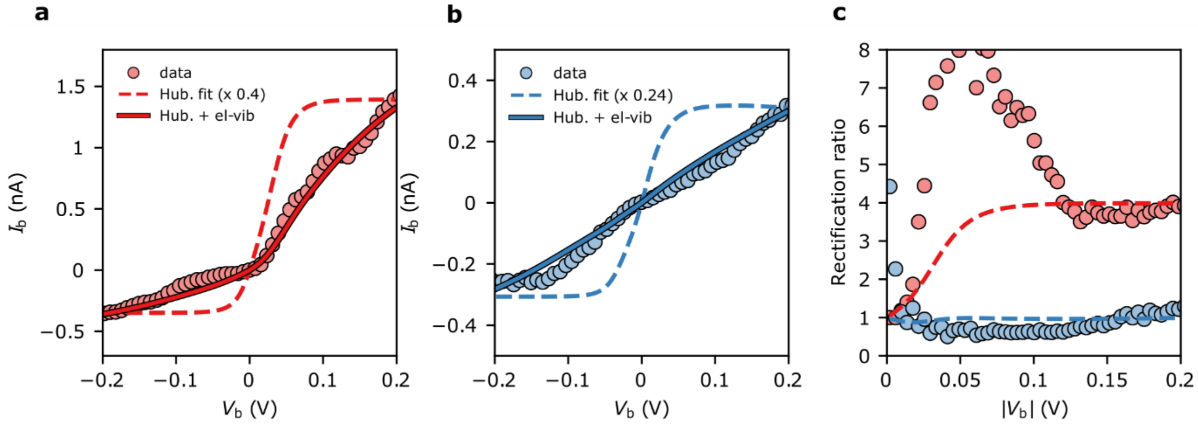


Figure S 6 (**a**) Device **B** *IV* traces on (**a**) the *N–4/N–3* resonance ($V_G$ = –21 V), and (**b**) the *N–3/N–2* resonance ($V_G$ = –11 V). The experimental data are plotted alongside *IV* traces taken from the Hubbard stability diagrams in Figure S5, and the Hubbard model plus electron-vibration coupling included in the electron transfer rates. Electron-vibration fitting parameters for *N–4/N–3*: $\Gamma_S = 5.9\ meV, \Gamma_D = 5.2\ \mu eV, \lambda_o = 66\ meV$; for *N–3/N–2* $\Gamma_L = 4.5\ meV, \Gamma_R = 2.1\ \mu eV, \lambda_o = 93\ meV$. (**c**) The rectification behaviour, $I_b(+V_b)$: $I_b(-V_b)$, of the *N–4/N–3* (red) and *N–3/N–2* (blue) transitions are given for the experimental values (circles) and the Hubbard model (dashed lines). The data was taken at a device temperature of 77 K.

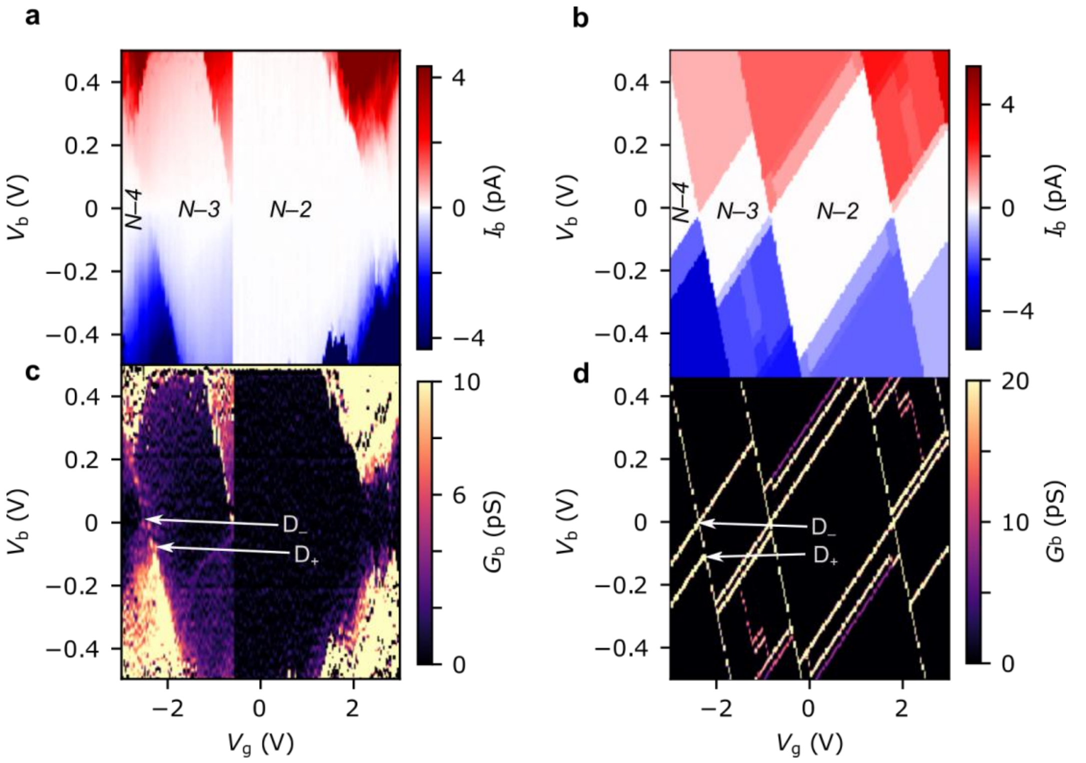


Figure S 7. Device **C**, measured at 4 K, experimental current (**a**) and conductance (**c**) stability diagrams. (**b**) and (**d**) are current and conductance stability diagrams calculated from the extended Hubbard model with parameters: $U$ = 0.49, $V$ = 0.19 and $t$ = –0.05 eV. The values of $t$, $\Gamma_S$ and $\Gamma_D$ were taken from the fit to the *N–3/N–4* IV trace in Figure S8. $\alpha_s$ and $\alpha_g$ were calculated from the gradients of the Coulomb diamond edges to be 0.23 and 0.18 respectively.

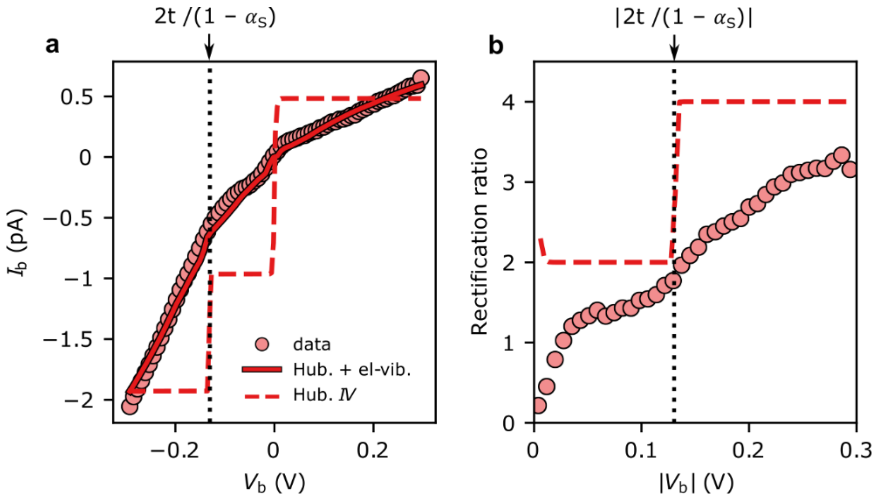


Figure S 8. (**a**) Device **C** *IV* trace on the *N–4/N–3* resonance ($V_G$ = –2.1 V). The experimental data (circles) are plotted alongside the *IV* traces taken from the Hubbard stability diagrams in Figure S7, and the Hubbard model plus electron-vibration coupling included in the electron transfer rates. The Hubbard *IV* is scaled by 0.4 for clarity. Electron-vibration fitting parameters for the trace are: $\Gamma_L = 7\ neV, \Gamma_R = 7.0\ meV, \lambda_o = 88\ meV$. (**b**) The rectification behaviour, $I_b(-V_b)$: $I_b(+V_b)$, of the *N–4/N–3* transition is given for the experimental values (circles) and the Hubbard model (dashed line). The data was taken at a device temperature of 4 K.

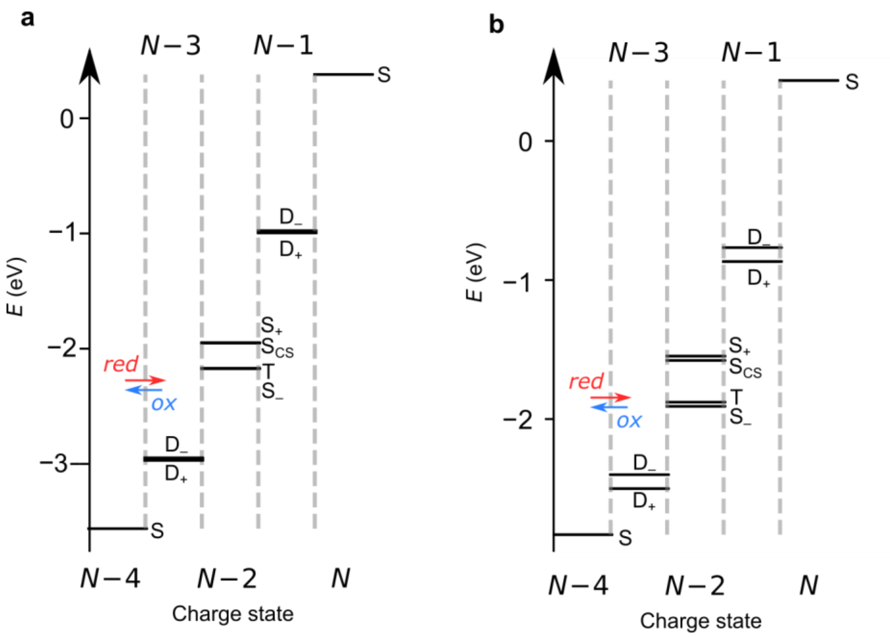


Figure S 9. Energy level diagrams of **FP3** in (a) device B and (b) device C at the lowest gate voltage in the stability diagrams show in Figure S5 and S7.

## Device A - Additional *IV* Fit

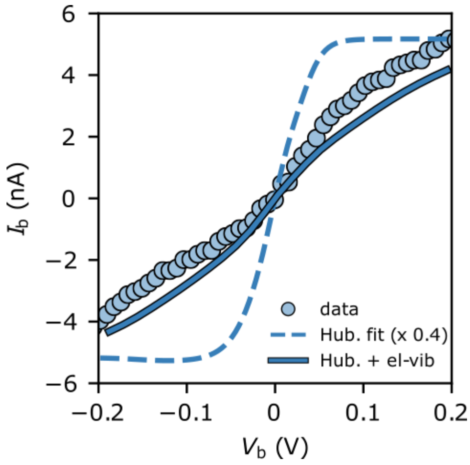


Figure S 10. (**a**) Device **A** *IV* trace on the *N–3*/*N–2* resonance. The experimental data and the Hubbard fit are the same data as in Figure 4b (main text). The Hubbard model plus electron-vibration coupling fit uses the electron-vibrational coupling as in Figure 4, but goes beyond the wide-band approximation by allowing the molecule-electrode coupling to vary from the *N–4*/*N–3* fit. The fitting parameters are slightly different: $\Gamma_S = 28\ \mu eV, \Gamma_D = 14\ meV, \lambda_o = 70\ meV$.

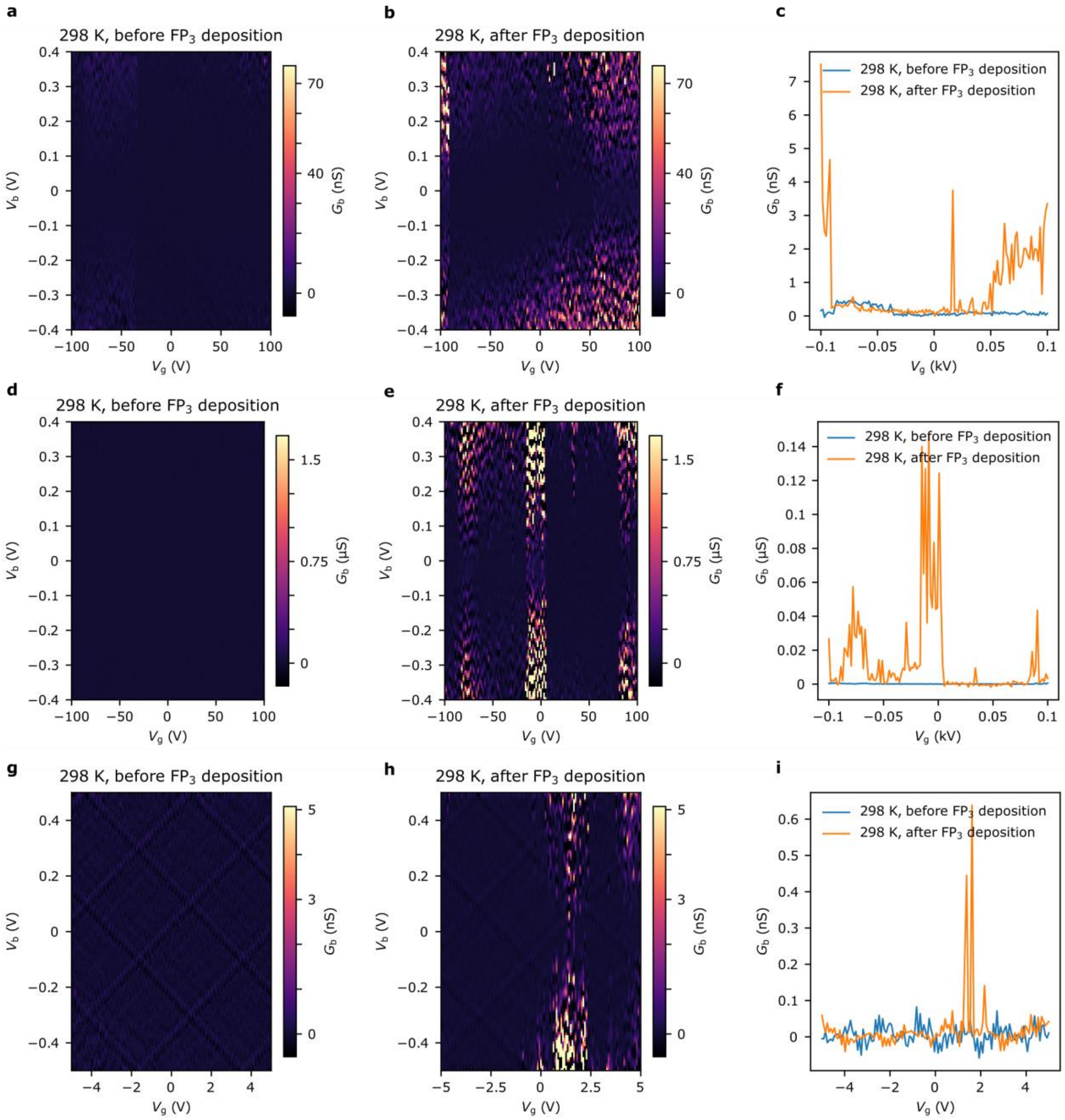


Figure S 11. Conductance stability diagrams taken before and after deposition of **FP3** onto the graphene nanogaps for devices **A** (**a, b**), **B** (**d, e**), and **C** (**g, h**). Conductance zero-bias gate traces before and after zero-bias gate traces for devices **A** (**c**) **B** (**f**), and **C** (**i**).

**Estimation of t from IV traces.**

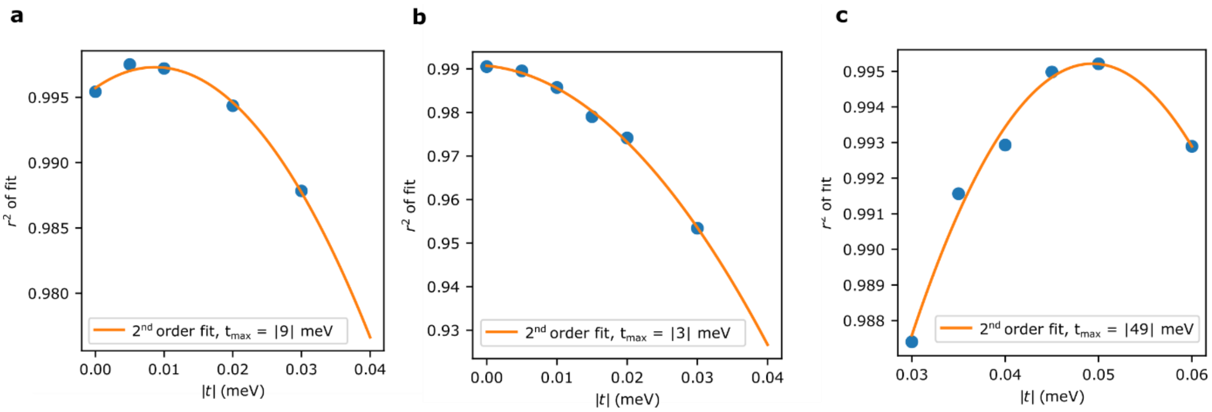


Figure S 12. $R^2$ of Hubbard + electron-vibration coupling fit to *N–4*/*N–3* resonant *IV* traces for devices **A**, **B** and **C** at different values of *t*. The values, combined with the stability diagrams give the estimation of *t*.

## Electron-Vibration Coupling

Electron-vibration coupling can be incorporated into the energy dependence of the electron-transfer rate constants, $k^{i \to j}$. For single-molecule junctions, the electron-transfer rate constants (assuming thermalized vibrations and the wide-band approximation) are given by:[3]

$$k(\epsilon) = \frac{1}{\pi} \mathrm{Re} \int_0^\infty e^{\rho i(\epsilon-\mu)t/\hbar} e^{-t/\tau} B(t) dt \quad (1)$$

where $\rho$ = +1 for reduction processes or –1 for oxidation and μ is the the chemical potential of the transition, and $\tau = 2\hbar/\Gamma$. The phononic correlation function, *B(t)* which can be thought of as a time-dependent Franck-Condon factor is given by:

$$B(t) = \exp\left[\int \frac{J(\omega)}{\omega^2}\left(\coth\left(\frac{\hbar\omega}{2k_B T}\right) \times (\cos\omega t - 1) - i\sin\omega t\right) d\omega\right] \quad (2)$$

As mentioned in the main text, the spectral density, $J(\omega)$, is constructed of two parts, $J(\omega) = \sum_q |g_q|^2 \delta(\omega - \omega_q) + J_{bg}(\omega)$, that account for the inner and outer sphere contributions to the rates of electron transfer. The electron-vibration coupling constants, $g_q$, are calculated from a DFT calculation from the optimized geometries and frequencies of **FP3** in the relevant charge states (as shown in the following section). The second term is the outer sphere contribution that corresponds to the reorganization energy of the substrate and wider local environment upon charging the molecule. This is modelled by phenomenologically by a superohmic spectral density function of the form:

$$J_{bg}(\omega) = \frac{\lambda_o}{2}\left(\frac{\omega}{\omega_c}\right)^3 e^{-\omega/\omega_c} \quad (3)$$

where $\lambda_o$ is the total outer sphere reorganization energy and $\omega_c$ is the cut-off frequency, (which is chosen to be 25 meV).[3]

For the *N–4/N–3* transition, the electron-transfer rates for reduction: $k^{S \to D+}$and $k^{S \to D-}$ (and similarly for oxidation, $k^{D+ \to S}$and $k^{D- \to S}$) are assumed to be the same except for an offset in energy by spacing between the doublets, *2t*. By making this assumption we take the geometric change that occurs upon oxidation to be greater than the geometric differences upon excitation from $D_+^{N-3}$ to $D_-^{N-3}$. Therefore the experimental *N–4/N–3 IV* traces undergo fitting with three free parameters, $\lambda_o, \Gamma_S$, and $\Gamma_D$.

The *N–3/N–2* resonant *IV* curve can also be fitted using this approach. The geometry of the *N–2* state (**FP3**$^{2+}$) is optimized by DFT in the singlet or triplet ground state. Therefore the $k^{D+ \to S_-}$ and $k^{D+ \to T}$ are evaluated separately. As with the *N–4/N–3* transition we assume the geometry of the doublets, $D_+^{N-3}$ and $D_-^{N-3}$ are the same. The transitions to/from the triplet are offset in energy by $E(T^{N-2}) - (S_-^{N-2})$, as calculated from the Hubbard model for each device. Therefore, as before, the fitting parameters are: $\lambda_o, \Gamma_S$, and $\Gamma_D$, and the electronic coupling to all states for the same charge transition are assumed to be the same.

## Calculation of Electron-Phonon Coupling Constants

Gaussian16[6] was used to carry out geometry optimisation and frequency calculations of **FP3** in charge (and spin) states: *N–4*, *N–3*, and *N–2* (singlet, S = 0) and *N–2* (triplet, S = 1). A B3LYP/6-31G(d) functional/basis set was used. Frequency calculations confirmed that each optimised geometry was an energy minimum. Electron-vibration coupling constants, $\Lambda_q$, for mode, $\omega_q$were calculated for each transition using a curvilinear coordinates system using the DUSHIN code,[7] and are displayed in Figure S13.

The total inner sphere reorganization energy, $\lambda_i$ of **FP3** upon electron transfer can be calculated as the sum of contributions from each mode:

$$\lambda_i = \hbar \sum_q \omega_q S_q \qquad (4)$$

where $S_q = \Lambda_q^2 = g_q/\omega_q$ is the Huang-Rhys parameter.

Inner-sphere reorganization energies for electron transfer were also calculated using the single-point energy calculations using the equation:

$$\lambda_i(SPE) = \frac{1}{2}\left(E_{N+1}(Q_N) - E_{N+1}(Q_{N+1}) + E_N(Q_{N+1}) - E_N(Q_N)\right) \qquad (5)$$

Where $E_N(Q_N)$ and $E_{N+1}(Q_{N+1})$ are the energies of the molecule in charge state *N* and *N+1* at their respective equilibrium geometries. $E_N(Q_{N+1})$ is the energy of the molecule in the *N* charge state but at equilibrium geometry of the *N+1* charge state. Similarly $E_{N+1}(Q_N)$ is the energy of the molecule in the equilibrium geometry of the *N* state, with *N+1* electrons.[8] The inner sphere reorganization energies calculated from both methods match well, and are displayed in Figure S14.

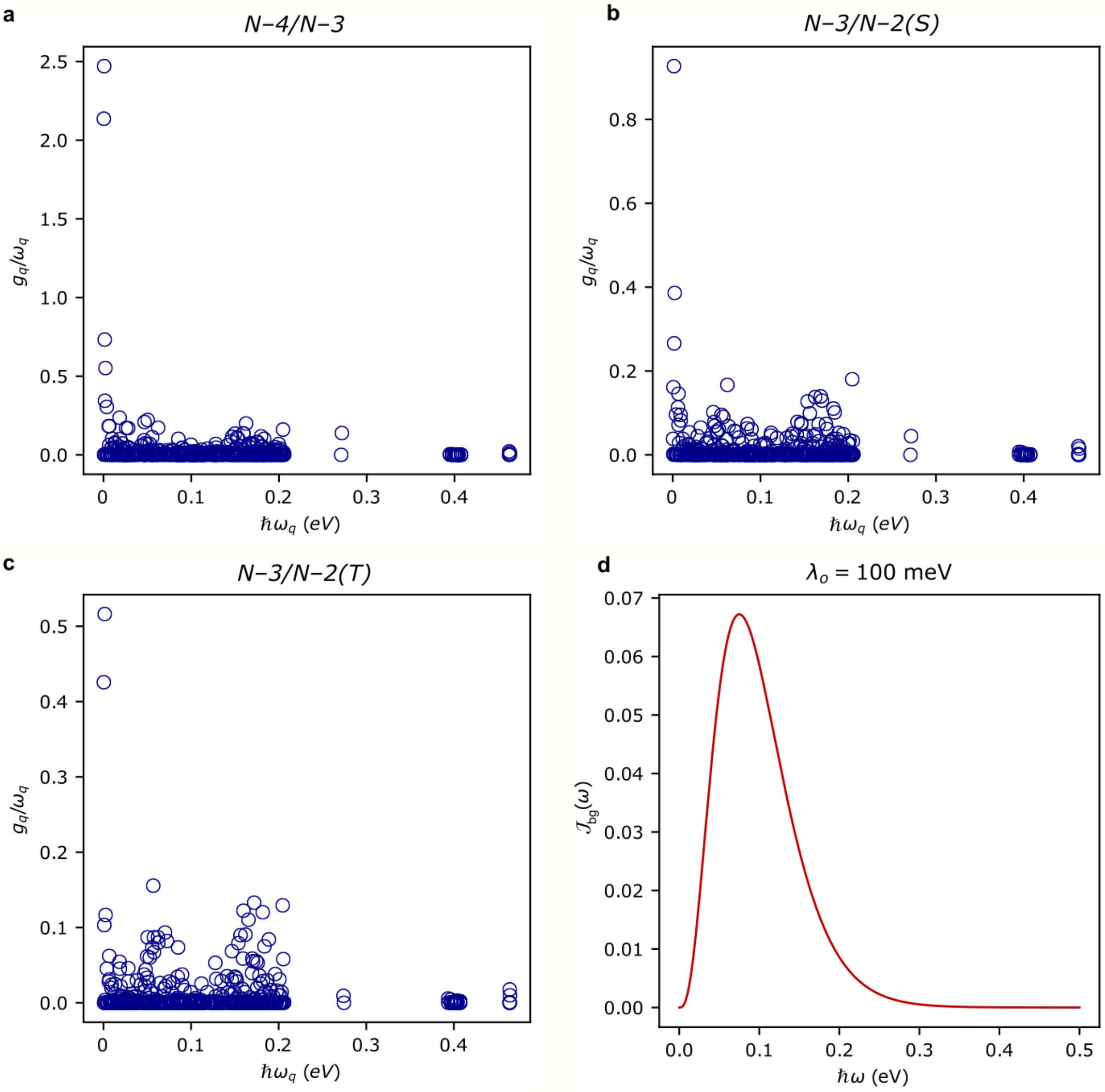


Figure S 13. DFT electron-vibration coupling constants for the (**a**) *N–4/N–3*, (**b**) *N–3/N–2*(S) and (**c**) *N–3/N–2*(T) transitions. (**d**) Background spectral density calculated for the parameters: $\lambda_o = 100$ meV, and $\omega_c$ = 25 meV.

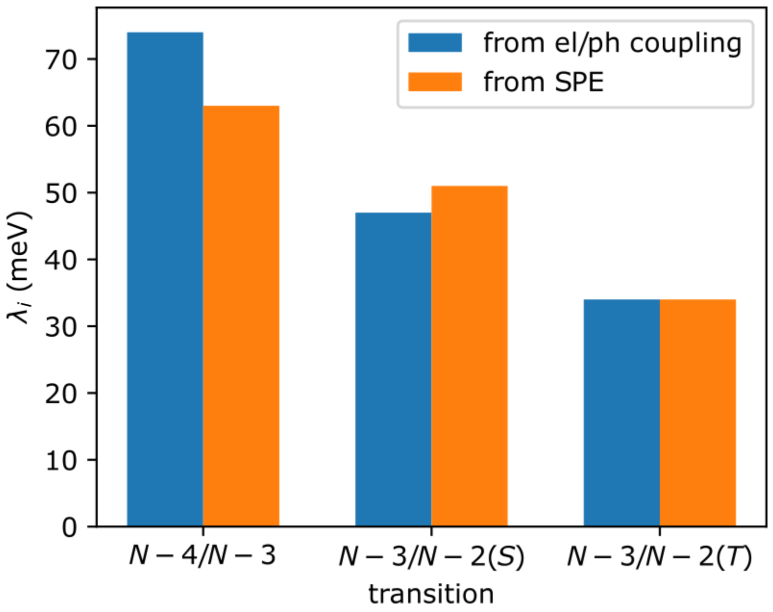


Figure S 14. DFT inner sphere reorganization energies, $\lambda_i$, for the transitions observed experimentally, as calculated from the sum of the contributions of each vibrational mode (blue) or from single-point energy (SPE) calculations.

**DFT calculation of *N–2* singlet-triplet energy spacing and *t***

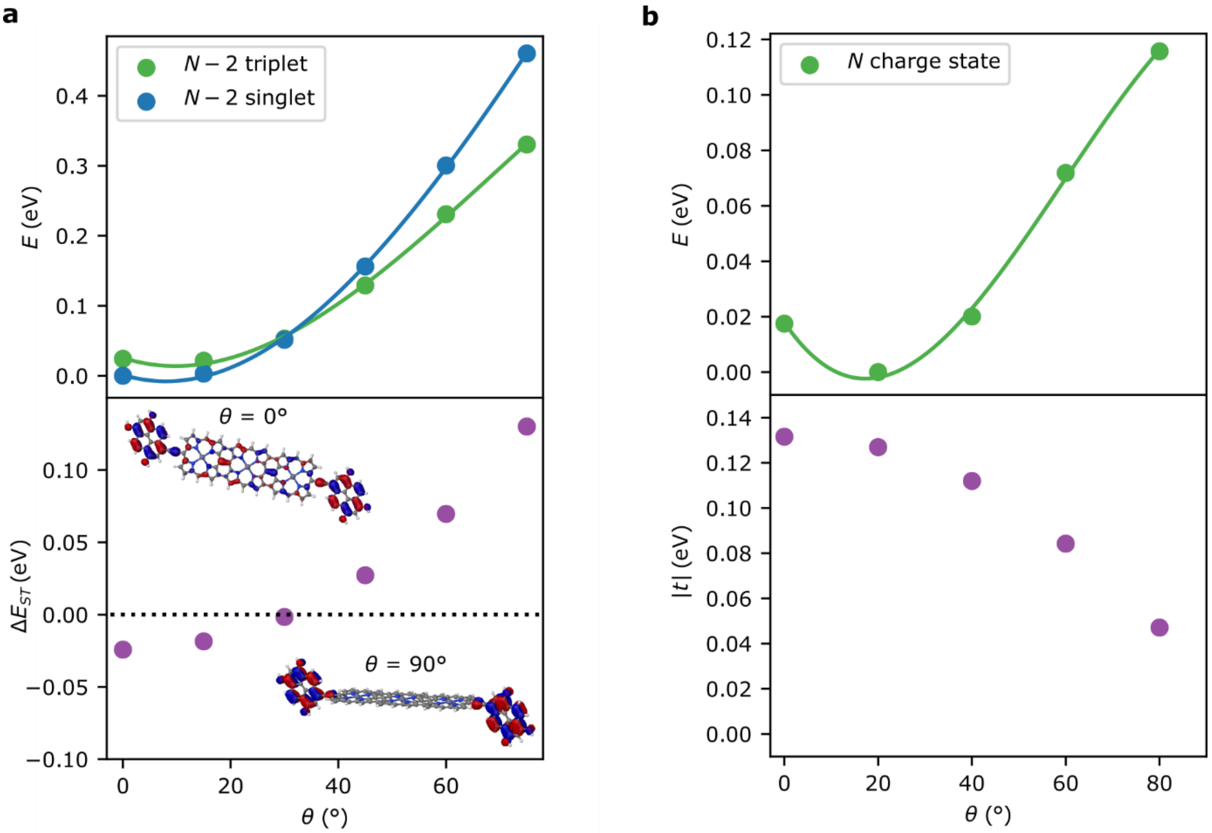


Figure S 15. (a) DFT-calculated relative energies of N–2 structures optimized as singlet and triplet spin states as a function of angle, $\theta$, between porphyrin trimer and anchor groups. Inset are MOs at the extremes for the singlet. The two states are in fact degenerate at $\theta \sim 30°$, therefore the splitting of only a few meV observed experimentally is a reasonable value. At $\theta > 30°$the wavefunction localises on the anchors and the overall ground state changes from singlet to triplet. (b) The energy of the N state as a function of $\theta$, and the calculation of t at each value, demonstrating the dependence on the molecular conformation. The distortion along the angle $\theta$ is one of many potential low-energy modes that the molecular can deform along upon adsorption onto the nano-gap.

## ESI References


1. S. Richert, B. Limburg, H. L. Anderson and C. R. Timmel, *J. Am. Chem. Soc.*, 2017, **139**, 12003-12008.
2. B. Limburg, J. O. Thomas, G. Holloway, H. Sadeghi, S. Sangtarash, I. C. Y. Hou, J. Cremers, A. Narita, K. Müllen and C. J. Lambert, *Adv. Funct. Mater.*, 2018, **28**, 1803629.
3. J. O. Thomas, B. Limburg, J. K. Sowa, K. Willick, J. Baugh, G. A. D. Briggs, E. M. Gauger, H. L. Anderson and J. A. Mol, *Nat. Commun.*, 2019, **10**, 4628.
4. F. Prins, A. Barreiro, J. W. Ruitenberg, J. S. Seldenthuis, N. Aliaga-Alcalde, L. M. K. Vandersypen and H. S. J. van der Zant, *Nano Lett.*, 2011, **11**, 4607-4611.
5. C. S. Lau, J. A. Mol, J. H. Warner and G. A. D. Briggs, *Phys. Chem. Chem. Phys.*, 2014, **16**, 20398-20401.
6. M. J. Frisch, G. W. Trucks, H. B. Schlegel, G. E. Scuseria, M. A. Robb, J. R. Cheeseman, G. Scalmani, V. Barone, G. A. Petersson, H. Nakatsuji, X. Li, M. Caricato, A. V. Marenich, J. Bloino, B. G. Janesko, R. Gomperts, B. Mennucci, H. P. Hratchian, J. V. Ortiz, A. F. Izmaylov, J. L. Sonnenberg, Williams, F. Ding, F. Lipparini, F. Egidi, J. Goings, B. Peng, A. Petrone, T. Henderson, D. Ranasinghe, V. G. Zakrzewski, J. Gao, N. Rega, G. Zheng, W. Liang, M. Hada, M. Ehara, K. Toyota, R. Fukuda, J. Hasegawa, M. Ishida, T. Nakajima, Y. Honda, O. Kitao, H. Nakai, T. Vreven, K. Throssell, J. A. Montgomery Jr., J. E. Peralta, F. Ogliaro, M. J. Bearpark, J. J. Heyd, E. N. Brothers, K. N. Kudin, V. N. Staroverov, T. A. Keith, R. Kobayashi, J. Normand, K. Raghavachari, A. P. Rendell, J. C. Burant, S. S. Iyengar, J. Tomasi, M. Cossi, J. M. Millam, M. Klene, C. Adamo, R. Cammi, J. W. Ochterski, R. L. Martin, K. Morokuma, O. Farkas, J. B. Foresman and D. J. Fox, *Journal*, 2016.
7. J. R. Reimers, *J. Chem. Phys.*, 2001, **115**, 9103-9109.
8. S. F. Nelsen, S. C. Blackstock and Y. Kim, *J. Am. Chem. Soc.*, 1987, **109**, 677-682.
